# A novel germline mutation in hMLH1 in three Korean women with endometrial cancer in a family of Lynch syndrome: case report and literature review

**DOI:** 10.1186/s13053-021-00185-y

**Published:** 2021-06-03

**Authors:** Youn-Joon Jung, Hye Ryoun Kim, Mi Kyung Kim, Eun-Ju Lee

**Affiliations:** 1 Department of Obstetrics and Gynecology, Chung-Ang University College of Medicine, 102 Heukseok-ro, Dongjak-gu, Seoul, 06973 South Korea; 2 Department of Laboratory Medicine, Chung-Ang University College of Medicine, 102 Heukseok-ro, Dongjak-gu, Seoul, 06973 South Korea; 3 Department of Pathology, Chung-Ang University College of Medicine, 102 Heukseok-ro, Dongjak-gu, Seoul, 06973 South Korea

**Keywords:** Lynch syndrome, C.1367delC mutation, Endometrial cancer, MLH1, Korea

## Abstract

**Background:**

Endometrial cancer is often the sentinel cancer in women with Lynch syndrome, among which endometrioid endometrial cancer is the most common. We found a Korean case of uterine carcinosarcoma associated with Lynch syndrome. And we reviewed 27 Korean women with endometrial cancer associated with Lynch syndrome already released in case report so far.

**Case presentation:**

The proband, a 45-year-old Korean woman received treatment for endometrioid adenocarcinoma. Her older sister and niece were treated for endometrioid adenocarcinoma and carcinosarcoma, respectively. Family history met the Amsterdam II criteria and immunohistochemical analysis revealed a loss of *MLH1* and *PMS2*. They all harbored a previously unreported germline likely pathogenic variant in c.1367delC in *MLH1*. They underwent staging operations including total hysterectomy, bilateral salpingo-oophorectomy, pelvic/paraaortic lymph node dissection, and washing cytology. All three women were healthy without evidence of relapse for over 4 years.

**Conclusion:**

This report indicates a novel germline c.1367delC variant in *MLH1*, and presents a Korean case of uterine carcinosarcoma associated with Lynch syndrome. Furthermore, the c.1757_1758insC variant in *MLH1* was suggested as a founder mutation in Lynch syndrome in Korean women.

## Introduction

Lynch syndrome, also known as hereditary non-polyposis colorectal cancer (HNPCC), is a hereditary cancer syndrome caused by germline mutations in DNA mismatch repair (MMR) genes. This condition is associated with malignant tumors in various extra-colonic sites including the uterus, ovaries, stomach, small intestine, urothelium, biliary tract, pancreas, brain, and skin. Uterine endometrial cancer is the second most common manifestation in HNPCC [1].

Tumorigenesis occurs owing to inactivating mutations in genes encoding essential proteins in the MMR pathway, which are primarily associated with the repair of replication errors. Human MMR genes including *MLH1*, *MSH2*, *MSH6, PMS1*, and *PMS2* are associated with this condition. In Lynch syndrome, approximately 50% of mutations occur in *MLH1*, 40% occur in *MSH2*, and 7% occur in *MSH6*.^2^ Fewer mutations have been reported in *PMS2* [2]. However, the prevalence of MMR gene mutations in Korean women, especially in endometrial cancer-associated Lynch syndrome, is unclear. Thus far, 20 mutations in MMR genes in 24 cases of Korean women with endometrial cancer associated with Lynch syndrome have been reported.

For effective preventive management, identification of families with Lynch syndrome is very important. Herein, we assessed a Korean family with Lynch syndrome and identified a novel germline *MLH1* variant*.* Moreover, this study summarizes germline variants in MMR genes in 27 Korean women with endometrial cancer associated with Lynch syndrome.

## Case report

**Fig. 1:**
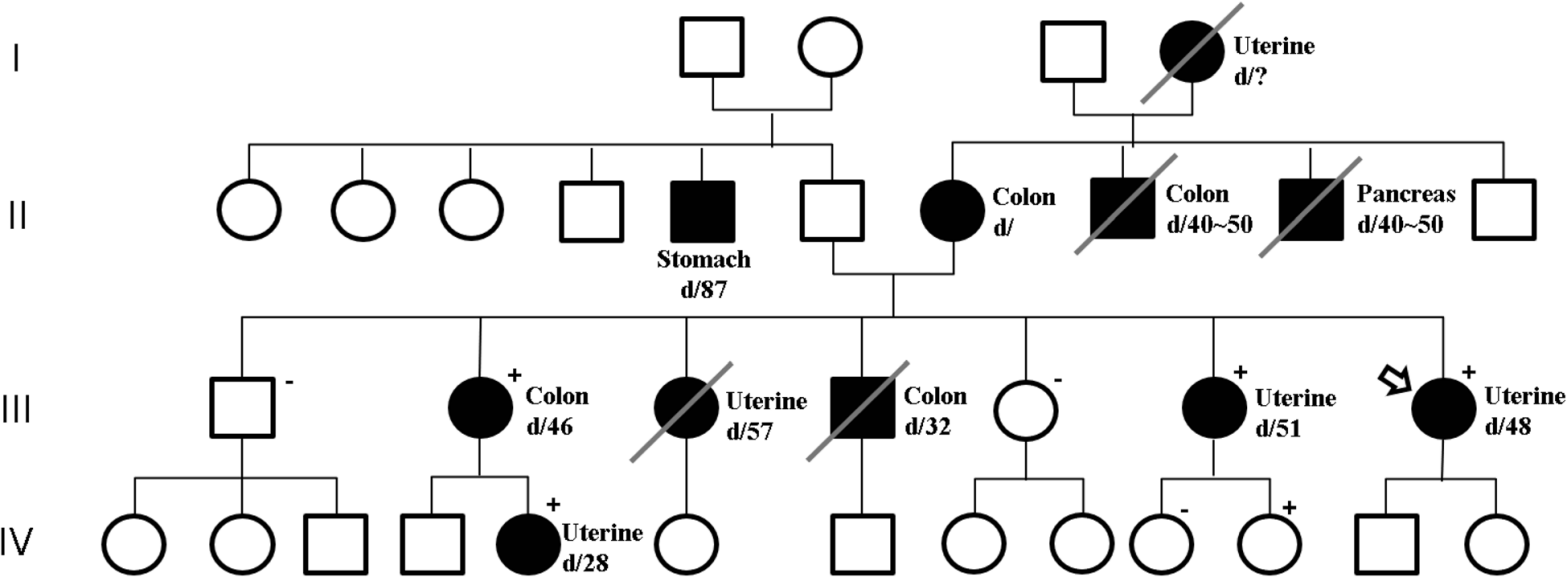
Pedigree of the family with Lynch syndrome. (Square, male; circle, female; oblique line, deceased;?, unknown; black, disease; d/number, diagnosed/age at diagnose; arrow, the proband; +, proven mutation carrier; −, non-mutation carrier) Not all relatives consented to undergo genetic screening

The proband marked III-7 (Fig. 1), a 45-year-old pre-menopausal woman and her elder sister, a 51-year-old premenopausal woman marked III-6 (Fig. 1), presented with abnormal vaginal bleeding. Pathological examination revealed endometrioid adenocarcinoma and imaging examination revealed that the disease was confined to the endometrial cavity. They underwent staging operations including total hysterectomy, bilateral salpingo-oophorectomy, pelvic/paraaortic lymph node dissection, and washing cytology. Her niece marked IV-5 (Fig. 1), a 28-year-old woman, was diagnosed with endometrial carcinoma and received 500 mg medroxyprogesterone acetate daily for 3 months to preserve fertility. However, the disease progressed and the aforementioned staging operations were performed for the niece. Histological examination revealed carcinosarcoma with lymph node metastasis. She received chemoradiation therapy with cisplatin-ifosfamide. All three women were healthy without evidence of relapse for over 4 years.

Their family history revealed a typical clustering of cancer. Five first- and second-degree relatives had also died of colon, pancreatic, or uterine cancer and four other relatives had also developed stomach, colon, or uterine cancer (Fig. 1). Ten of her relatives were diagnosed with Lynch syndrome-associated cancers across four generations. Therefore, this family meets the Amsterdam II criteria.

**Fig. 2:**
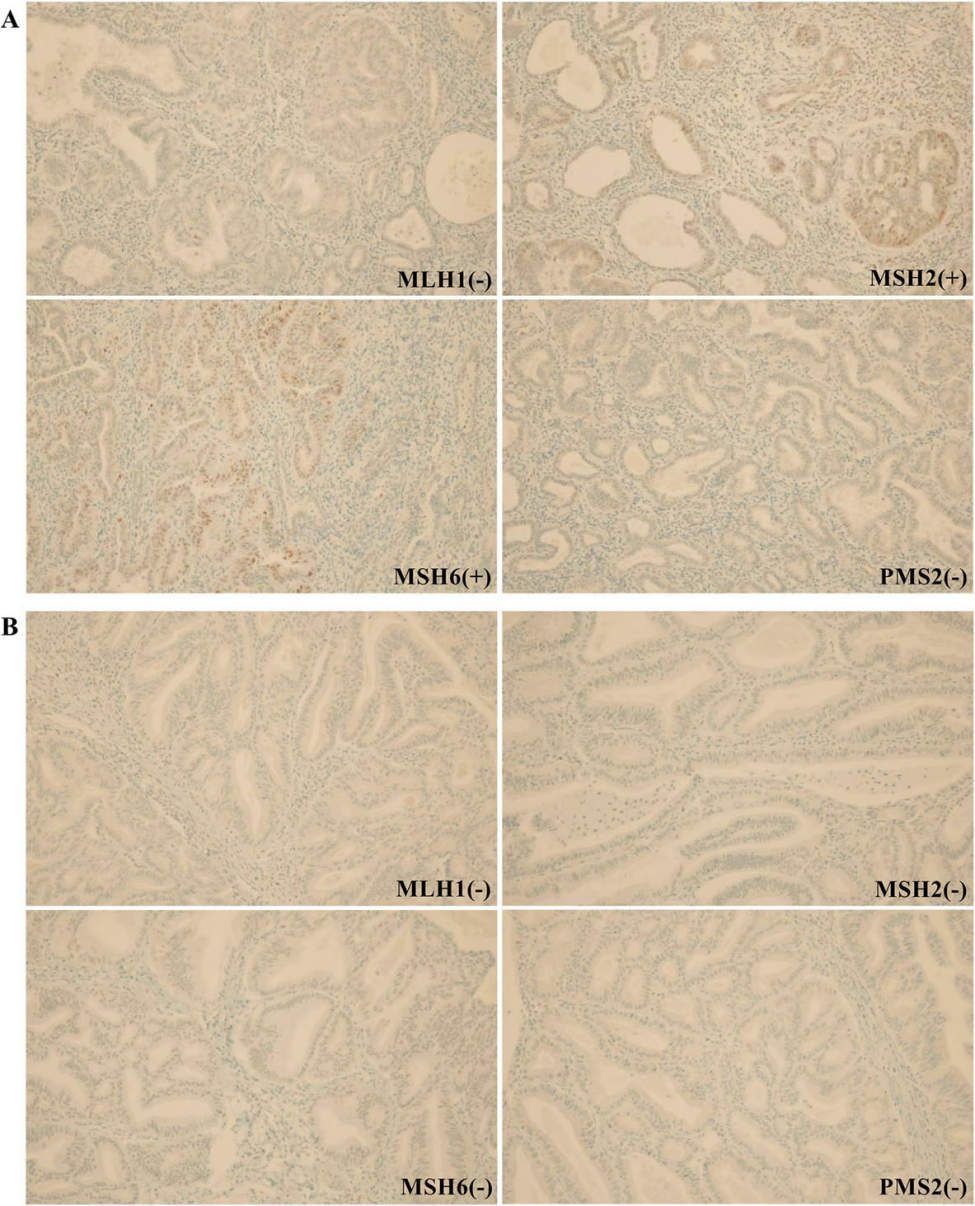
Analysis of mismatch repair genes in the proband and proband’s elder sister. **A** Proband’s immunohistochemical analysis for *MLH1*, *MSH2*, *MSH6*, and *PMS2* revealed negative staining for *MLH1* and *PMS2*, representing the loss of expression. **B** Results of immunohistochemical analysis of the proband’s elder sister showed negative staining for *MLH1*, *MSH2*, *MSH6*, and *PMS2*

**Fig. 3:**
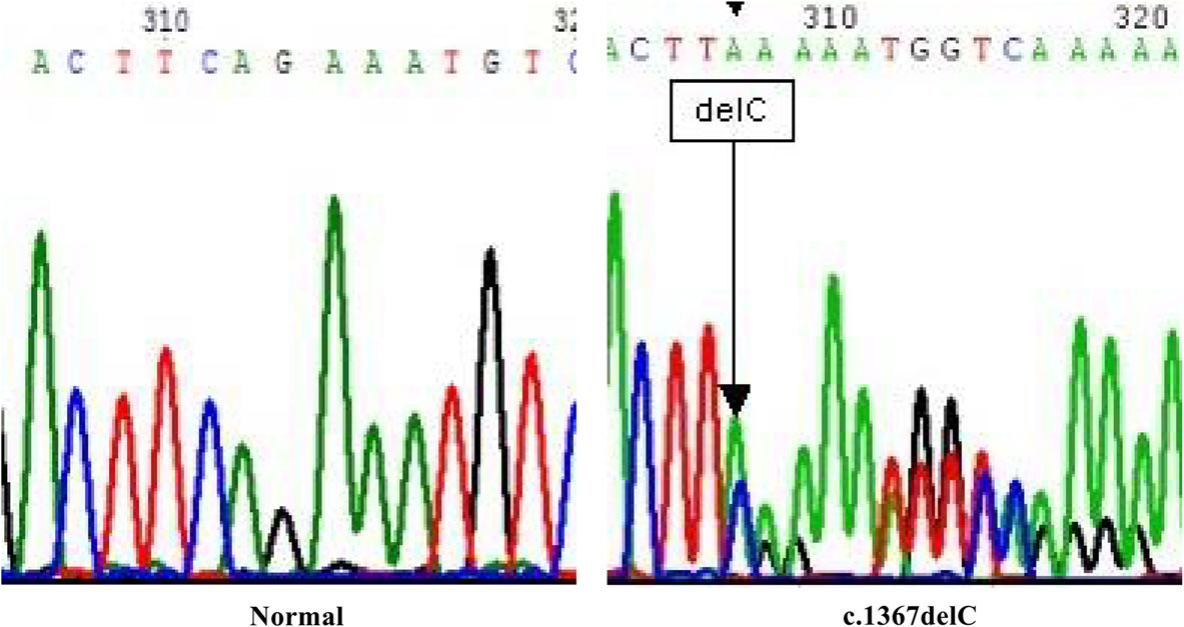
Analysis of mismatch repair genes in the proband. Gene sequencing revealed the c.1367delC mutation in *MLH1*

Immunohistochemical analysis of MMR protein was performed in biopsy specimens of patients. MLH1 and PMS2 expression were negative in the proband shown and her niece, indicating the loss of these proteins (Fig. 2a). Furthermore, MLH1, MSH2, MSH6, and PMS2 were deficient in the proband’s elder sister (Fig. 2b). To identify Lynch syndrome, *MLH1* (reference sequence: NM 000249) and *MSH2* (reference sequence: NM 000251) variants were evaluated using genomic DNA from peripheral blood of the proband. Polymerase chain reaction (PCR) amplification and Sanger sequencing were performed for genetic analysis (Seoul clinical laboratories, ABI veriti for PCR amplification, ABI 3500DX for Sanger sequencing). A c.1367delC variant was detected in *MLH1* (Fig. 3); however, no variant was detected in *MSH2*. We performed genetic analyses for the *MLH1* variant among the proband’s relatives and III-2, III-6, IV-5, and IV-11 displayed the same *MLH1* variant (Fig. 1).

## Discussion

Herein, we report the case of three Korean women with endometrial malignancy from a family with Lynch syndrome. This study is the first to report the c.1367delC variant in *MLH1* in Lynch syndrome. Moreover, one of three women had uterine carcinosarcoma, which, to our knowledge, is a novel finding in relation to Lynch syndrome in Korean women.

This c.1367delC variant corresponds to a likely pathogenic variant according to standards and guidelines of American College of Medical Genetics and Genomics for the interpretation of sequence variants. For the c.1367delC variant to be classified as pathogenic, further studies such as functional analysis are needed.

We reviewed literatures and summarized germline mutations in MMR genes in 27 Korean women with endometrial cancer associated with Lynch syndrome, including the present three cases (Table 1) [1, 3–13]. The average diagnostic age for endometrial cancer was 46.3 years. The *MLH1* mutation was the primarily harbored mutation (51.9%, *n* = 14), other mutations included those in *MSH2* (37.0%, *n* = 10) and *MSH6* (11.1%, *n* = 3). These mutation rates are concurrent with those reported previously for Lynch syndrome in Caucasian individuals (*n* = 448) [2]. However, compared with the mutations rates for colorectal cancers associated with Lynch syndrome in 44 Korean individuals, wherein only four patients with endometrial cancers were included and reported mutation rates of 70.5, 22.7, and 6.8% in *MLH1*, *MSH2*, and *MSH6*, respectively [1], the *MLH1* mutation rate is greater in colon cancer than in endometrial cancer associated with Lynch syndrome.

**Table 1:** Germline mutation in DNA mismatch repair genes in Korean women with endometrial cancer associated with Lynch syndrome

Gene	Exon	Nucleotide changes	age^**a**^	Amsterdam^**b**^	Histology^**c**^	Grade	FIGO stage	MSI	Loss of MMR^**d**^	Cancer	Reference
*MLH1*	11	c.1008delG	37	Yes	–	–	–	–	–	E,C	3
*MLH1*	12	c.1367delC	45	Yes	Endometrioid	1	IA	–	MLH1, PMS2	E	Present
*MLH1*	12	c.1367delC	50	Yes	Endometrioid	2	IB	–	MLH1, MSH2, MSH6, PMS2	E	Present
*MLH1*	12	c.1367delC	27	Yes	Carcinosarcoma	3	IA	–	MLH,1 PMS2	E	Present
*MLH1*	15	c.1721 T > C	33	Yes	–	–	–	–	–	E,C,L	3
*MLH1*	16	c.1757_1758insC	45	Yes	Endometrioid	–	–	MSI-H	MLH1	E, C	4
*MLH1*	16	c.1757_1758insC	56	No	–	–	–	MSS	MLH1	E	5
*MLH1*	16	c.1757_1758insC	35	No	–	–	–	–	–	E	6
*MLH1*	16	c.1780_1781insC	55	Yes	–	–	–	–	MLH1	E	7
*MLH1*	16	c.1780_1781insC	46	Yes	–	–	–	–	MLH1	E,C	7
*MLH1*	16	c.1780_1781insC	45	Yes	–	–	–	–	MLH1	E,C	7
*MLH1*	16	c.1878_1881del	53	No	Endometrioid	–	–	–	MLH1	E,C,S	8
*MLH1*	17	c.1907 T > C	38	Yes	–	–	–	–	–	E,C,Sb	1
*MLH1*	19	c.4484 T > A	57	No	Endometrioid	–	–	MSI-H	MLH1	E, C	4
*MSH2*	1	c.23C > T	55	No	Endometrioid	1	IA	MSI-H	MSH2	E	9
*MSH2*	5	c.882delT	42	Yes	–	–	–	–	–	E	6
*MSH2*	IVS5	c.942 + 2dupT(IVS5)	47	Yes	Endometrioid	–	–	MSI-H	MSH2	E, C, Cx	4
*MSH2*	12	c.1886A < G	40	No	–	–	–	–	–	E	6
*MSH2*	12	c.1886A > G	49	Yes	Endometrioid	1	IA	MSI-H	MSH2	E,C	10
*MSH2*	13	c.2089 T > C	44	Yes	–	–	–	MSI-H	MSH2, MSH6	E	5
*MSH2*	13	c.2184_2186dup	51	Yes	Dedifferentiated	3	–	MSI-H	Normal^e^	E,C	8
*MSH2*	15	c.2633_2634delAG	35	Yes	–	–	–	–	–	E,C	11
*MSH2*	7	c.2634_2634 + 1delGg	43	Yes	–	–	–	MSI-H	MLH1, MSH2	E	5
*MSH2*	16	c.2649 T > G	41	Yes	Endometrioid	1	II-	–	–	E,C	12
*MSH6*	5	c.3206G > A	72	No	–	–	–	MSI-H	MSH6	E	5
*MSH6*	5	c.3261dupC	60	Yes	Serous	–	IA	–	MLH1	E, C	13
*MSH6*	9	c.3823G > A / c.3821_3824dupAATG	48	No	–	–	–	MSI-H	MSH2, MSH6	E	5

*E* endometrial cancer, *C* colorectal cancer, *L* Liver cancer, *Sb* small bowel cancer, *S* stomach cancer, *Cx* cervical cancer, *MMR* Mismatch repair, *MSS* microsatellite stability, *MSI* microsatellite instability

^a^Age at diagnosis of endometrial cancer; ^b^Meeting Amsterdam II criteria; ^c^Histologic type of endometrial cancer; ^d^evaluated with immunohistochemical analysis; ^e^Only MLH1 and MLH2 were evaluated via immunohistochemical analysis and yielded positive results

The c.1367delC variant in *MLH1* in this family has not been previously reported in studies published in English according to our literature survey. Herein, the two variants c.1367delC and c.1780_1781insC in *MLH1* were respectively harbored in three women belonging to the same family. The c.1757_1758insC variant in *MLH1* being the most common in the three family members (Table 1) and occurring commonly in colorectal cancer associated with Lynch syndrome (11 of 31 families) [1]. Together, the c.1757_1758insC variant in *MLH1* could be suggested to be a founder mutation in Lynch syndrome in Korean individuals rather than in individuals in other countries.

Despite the controversy regarding the dominant histologic subtype of endometrial cancer in Lynch syndrome, endometrioid adenocarcinoma is most frequent [14], and a few cases of nonendometrioid adenocarcinoma have been reported [15]. In the present literature review, histological characteristics were determined in 12 cases and three women had non-endometrioid type tumors including serous adenocarcinoma, dedifferentiated adenocarcinoma, and carcinosarcoma. This is the first report of a case of carcinosarcoma associated with Lynch syndrome in Korean women.

In conclusion, this study reports a novel likely pathogenic variant c.1367delC in *MLH1* in Lynch syndrome and the case of Korean women with uterine carcinosarcoma associated with Lynch syndrome.

## Data Availability

Data generated or analysed during this study are available from electronic medical record of Chung-Ang university hospital. But restrictions apply to the availability of these data, which were used under license for the current study, and so are not publicly available. The data are however available from the authors upon reasonable request and with permission of Chung-Ang university hospital.

## Abbreviations

HNPCC: Hereditary non-polyposis colorectal cancer
MMR: Mismatch repair
PCR: Polymerase chain reaction
